# The Relationship of Acculturation, Traumatic Events and Depression in Female Refugees

**DOI:** 10.3389/fpsyg.2020.00906

**Published:** 2020-05-14

**Authors:** Annabelle Starck, Jana Gutermann, Meryam Schouler-Ocak, Jenny Jesuthasan, Stephan Bongard, Ulrich Stangier

**Affiliations:** ^1^Clinical Psychology and Psychotherapy, Department of Psychology, Goethe-University Frankfurt, Frankfurt, Germany; ^2^Psychiatric University Clinic of Charité at St. Hedwig Hospital, Berlin, Germany

**Keywords:** female refugees, traumatic events, depressive symptoms, acculturation, integration, marginalization

## Abstract

Recent research has identified significant correlations between traumatic events and depression in refugees. However, few studies have addressed the role of acculturation strategies in this relationship. This study explored the relationship between cultural orientation, traumatic events and depression in female refugees from Syria, Afghanistan, Eritrea, Iran, Iraq, and Somalia living in Germany. We expected acculturation strategies to moderate the effect of traumatic experiences on depression. The sample included 98 female refugees in Germany. The depression scale of the Hopkins Symptom Checklist (HSCL) represented the dependent measure. The trauma checklists derived from the Post-traumatic Diagnostic Scale (PDS) and the Harvard Trauma Questionnaire (HTQ) as well as the Frankfurt Acculturation Scale (FRACC) were used as independent measures for traumatic events and *orientation toward the host culture* as well as *orientation toward the culture of origin*, respectively. A moderation analysis was conducted to examine whether the relationship between the number of traumatic events and depression was influenced by the women’s *orientation toward the culture of origin* and the *host culture*. We identified a significant model explaining 26.85% of the variance in depressive symptoms (*Cohen’s f^2^* = 0.37). The number of traumatic events and the *orientation toward the host culture* exerted significant effects on depressive symptoms. The moderating effect was not significant, indicating that the effect of the number of traumatic events was not influenced by cultural orientation. Based on our results, *orientation toward the host culture* as well as traumatic experiences exert independent effects on depressive symptoms in refugees.

## Introduction

Refugees arriving in Germany are often burdened by the impact of traumatic events. Life-threatening attacks, violent deaths of relatives and permanent danger in daily life in war zones are the most frequently reported traumatic experiences (Richter et al., 2015; Jesuthasan et al., 2018). In particular, 41% of Middle Eastern refugees have experienced violent attacks, and 87% report war experiences (Kröger et al., 2016). Among Arabic-speaking asylum seekers in Germany, 80% reported having personally experienced or witnessed traumatic events (Georgiadou et al., 2017).

The mental health of women is often reported to be more threatened than men’s mental health (Rasmussen et al., 2014). Although women are rarely on the frontlines of war, they face severe threats such as domestic violence, rape, sex trafficking, and different types of sexual torture (Hynes, 2004; Berman et al., 2006), often resulting in rejection by the family or community (Sideris, 2003). Gender-specific trauma also includes forced sterilization or abortion, forced marriage, or widespread practice such as genital mutilation, and punishments such as dowry killing (Mogga, 2017; Jesuthasan et al., 2018).

In general, exposure to traumatic events increases the risk of developing not only post-traumatic stress disorder (PTSD) but also depression (Price and van Stolk-Cooke, 2015). This increased risk is reflected in the high proportion of Middle Eastern refugees showing depressive symptoms (Kröger et al., 2016). Point prevalence rates for depression vary between studies, ranging from 37 to 44% for Syrian refugees (Naja et al., 2016; Acarturk et al., 2018) and 28 to 75% for Iraqi refugees (Slewa-Younan et al., 2012). In one review, the reported rates for point prevalence of depression was even higher than for PTSD (Slewa-Younan et al., 2015). Furthermore, the accumulation of traumatic experiences is associated with increased severity of depression (Tracy et al., 2014; LeMaster et al., 2017).

Depression is predicted not only by the accumulated experience of potentially traumatic events but also by post-migration stressors (Dulin and Passmore, 2010; Boettche et al., 2016). Thus, the strength of the relationship between the number of traumatic events prior to migration and depression remains unclear (Hexel and Sonneck, 2002; Tanskanen et al., 2004; Heeren et al., 2012). However, stressful life events in general may lead to depression (Kendler and Gardner, 2016), and traumatic events predict depression in migrants (Ward et al., 2018).

The results from several studies also suggest potential changes in the association between the number of traumatic events and depression over time (Phillips et al., 2015). In a cross-sectional study, the correlation between the number of traumatic events and depression was higher in asylum seekers who had just arrived in the host country than in individuals who had lived in the host country for 12 to 26 months (Heeren et al., 2012). Thus, in addition to uncertainty about their asylum status and future, long-term distress associated with adaptation to new living conditions may contribute to the development of depression among refugees.

Processes of adaptation from the culture of origin to a new, receiving culture are referred to as acculturation. Acculturation strategies are defined as principally independent dimensions of *orientation toward the culture of origin* and the *receiving culture*. A common classification of acculturation strategies was proposed by Berry (1997), who distinguished *assimilation* (high *orientation toward the host culture* but low *orientation toward the culture of origin*), *separation* (*high orientation toward the culture of origin and low orientation toward the host culture*), *integration* (*high orientation toward* both the *host culture* and *culture of origin* simultaneously) and *marginalization* (low *orientation toward* both *the host culture* and *culture of origin* simultaneously).

According to Berry’s (1997) model of acculturation, predominant acculturation strategies influence psychological well-being. Acculturation strategies have also shown to influence different aspects of mental health (Yoon et al., 2013) such as anxiety (Ince et al., 2014) or depression (Nakash et al., 2015; Lincoln et al., 2016). They are also postulated to incorporate other unique protective factors as well (Berry, 1997).

The acculturation process itself appears to differ between genders. Although male Iranian refugees in Sweden and Sudanese refugees in Australia appear to engage more in *separation*, e.g., resisting the new culture and maintaining their traditional authoritarian roles, females tend to adopt new values (Darvishpour, 2002; Khawaja and Milner, 2012). Again, certain challenges appear to be specific to women. For example, for women from societies with traditional gender hierarchies, a dislocation from group or family support is often associated with high social and individual costs (Guzder, 2011). Additionally, liberties provided to women in host cultures might cause marital stress (Khawaja and Milner, 2012). These female-specific challenges, as well as the distress associated with acculturation processes (Berry and Annis, 1974; Williams and Berry, 1991), can lead to depressive symptoms (Liu et al., 2016; Singh et al., 2017).

The challenges associated with acculturation and acculturation itself have been shown to influence depression (Ji and Duan, 2006). For instance, a higher intensity of identification with the host culture significantly predicted a reduction in depressive symptoms (among Asian Indian international students; Meghani and Harvey, 2016). Moreover, migrants’ participation in the host culture is associated with a lower depression risk (Ince et al., 2014). However, *orientation toward the host culture* predicts depression only when *orientation toward the culture of origin* was moderate to high (Yu et al., 2016). Protective effects of culture of origin have been identified; for example, the so called Hispanic paradox defined as the condition in which Mexican Americans show a level of mental health comparable to their Caucasian peers, despite a disadvantaged socioeconomic status (Markides and Coreil, 1986). Similarly, Latino immigrants with a greater orientation toward mainstream American culture experience more depressive symptoms (Torres, 2010). Additionally, for East-African migrants in Germany, maintaining a strong *orientation toward the culture of origin* is associated with less drug abuse (Bongard et al., 2015). Finally, cultural attachment to culture of origin measured using implicit measures appears to be associated with increased resilience (Hong et al., 2013). Thus, a study considering the interactions of both dimensions represented in acculturation strategies is important. A meta-analysis found evidence that *integration* is associated with many advantages concerning mental health outcomes (Yoon et al., 2013). Specifically regarding depressive symptoms, *integration* is associated with a lower risk of depression, and *separation* is associated with a higher risk (Ince et al., 2014). In other studies, a low *orientation toward the culture of origin* and toward the *receiving culture*, i.e., *marginalization*, is associated with higher levels of depression (Choi et al., 2009; Kim, 2009; Kupper et al., 2018).

Since traumatic experiences and acculturation processes might affect depressive symptoms (Oppedal and Idsoe, 2012; LeMaster et al., 2017), the interaction between trauma and acculturation strategies might be a crucial factor. Consistent with this assumption, a previous study (Ngo et al., 2001) observed variation in the effect of pre-migration traumatic experiences on depression in migrants according to the level of acculturation. Traumatic experiences exerted a stronger effect on depressive symptoms in migrants with lower acculturation levels. However, this study ignored predominant acculturation strategies and defined acculturation only in terms of language use. Researchers have not clearly determined whether this relationship is also replicated in refugees.

## The Present Study

The aims of the present study were first to investigate whether depressive symptoms in a specific population of female refugees from Syria, Afghanistan, Eritrea, Iran, Iraq, and Somalia were related to the number of traumatic events the refugees experienced. Second, because previous research has reported that acculturation also affects depression (Yoon et al., 2013), we examined whether this relationship was moderated by *orientations toward the host culture* as well as the *culture of origin*. Unlike previous studies, in which acculturation has not been theoretically explicitly defined, in this study, Berry’s acculturation theory (Berry, 1997) served as a basis to investigate the effect of the acculturation strategy adopted by the refugees.

Since *integration* may function as a protective factor (Yoon et al., 2013; Ince et al., 2014) whereas *marginalization* may function as a risk factor (Choi et al., 2009; Kim, 2009) we expected that in our group of female refugees, cultural orientation would moderate the relationship of traumatic events on depression. Firstly, we expected *orientation toward the host culture* and *orientation toward the culture of origin* to be moderating variables. Secondly, we hypothesized that the interaction of both, the *orientation toward the host culture* and *the culture of origin*, representing acculturation strategies would moderate the effect of number of traumatic events on depression as well. Thereby we expected *integration* (i.e., *high orientation toward* both the *host culture* and *culture of origin* simultaneously) to buffer the effect of the number of traumatic events on depressive symptoms and that the number of traumatic events would have a greater influence on depression in female refugees using *marginalization* (i.e., *low orientation toward* both the *host culture* and *culture of origin* simultaneously) than women with other predominant acculturation strategies.

## Materials and Methods

### Procedure

The current study was part of a multicenter study called the “Female Refugee Study” (Jesuthasan et al., 2018) conducted in five German federal states (Berlin, Bavaria, Mecklenburg-Western Pomerania, Hesse and Rhineland-Palatinate). Ethical approval was obtained from the Ethics Committee 1 of Charité Berlin (EA1/117/16) as well as the University Hospital Frankfurt (334/16). A sample of female refugees from Syria, Afghanistan, Eritrea, Iran, Iraq, and Somalia was recruited according to the likelihood of a successful decision on their asylum applications and granting of their refugee status as well as the quota for countries of origin of the refugees in Germany. Only women aged 18 years or older who were living in randomly selected shared accommodations were included.

Recruitment occurred via open briefings at the accommodation locations and in person. Participants received both written and verbal information about the study, and written informed consent was obtained by native speakers of the participants’ primary language at least 24 h before the interview began. These native speakers also conducted the interviews. The women were able to withdraw their participation at any time without giving reasons and without any costs or disadvantages.

The data were collected in face-to-face interviews conducted by trained female interviewers speaking the native language of the female refugees. They had at least a bachelor’s degree in psychology or medicine or related subjects (orient studies, communication). A 2-day training session and an additional 1-day training included general and specific interview skills and prepared the interviewers also to work with potentially traumatized women. Supervision and weekly team meetings were provided throughout the study. Furthermore, clinically experienced clinicians were available on-call throughout the study.

All measures were translated into Arabic, Farsi, Somali, and Tigrinya. A process of translation and retranslation ensured translation quality. If required, consultations by native-speaking psychologists or interpreter-aided consultations were offered. The interviews were conducted between August and December 2016.

### Participants

The initial sample included 106 women living in Frankfurt. Eight women were excluded because more than 30% of their values were missing. If necessary, the women were offered an opportunity to visit our counseling center. For women in unstable situations, it did not seem appropriate to push them to talk if they did not want to; therefore, we did not question the participants intensively if they refused to respond to some questions. Nevertheless, this approach resulted in missing data and may have caused an under- or overestimation of some results (Jesuthasan et al., 2018).

The following results relate to the final sample of 98 women. The mean age of the participants was 29.3 years (*SD* = 8.7), and their ages ranged from 18 to 61 years. Forty-six participants (46.9%) spoke Farsi as their mother tongue, 37.8% (*n* = 37) spoke Arabic, 14.3% (*n* = 14) spoke Tigrinya and 1% (*n* = 1) spoke Somali. This language distribution was mainly consistent with the nationalities of the women: 31.6.1% (*n* = 31) were from Afghanistan, 27.6% (*n* = 27) were from Syria, 14.3% (*n* = 14) were from Eritrea, 9.2% (*n* = 9) were from Iran, 8.2% (*n* = 8) were from Iraq, 1.0% (*n* = 1) were from Somalia, and 7.1% (*n* = 7) had other nationalities (Tajik and Turkmen); one participant did not respond to the question about nationality. Regarding marital status, 68.4% (*n* = 67) of the women were married and living with their partners, and 77.6% (*n* = 76) had children. Concerning the level of education, 17.3% (*n* = 17) had not gone to school, 49.0% (*n* = 48) had gone to school, 19.4% (*n* = 19) had started or finished vocational training or study, and the data were missing for 14.3% (*n* = 14) of the participants. The reported religions were as follows: 74.5% (*n* = 73) Islam, 14.3% (*n* = 14) Christian, 1.0% (*n* = 1) Yazidi, 2.0% (*n* = 2) other, 2.0% (*n* = 2) none, 5.1% (*n* = 5) did not want to specify a religion, and 1.0% (*n* = 1) did not respond. The mean duration since the women had left their homes was 34.3 months (*SD* = 55.5). The mean duration of time since they had registered as seeking asylum was much shorter, namely, 11.9 months (*SD* = 6.8), which might have been due to a long duration of travel.

### Measures

#### Frankfurt Acculturation Scale (FRACC)

The Frankfurt Acculturation Scale (FRACC) is based on Berry’s model of acculturation (Bongard et al., 2007, 2020). It consists of two aspects of acculturation: the “*orientation toward the culture of origin*/OC,” e.g., “I live close to the traditions of my country of origin,” which had a Cronbach’s α = 0.72 in previous research (Morawa and Erim, 2014; Cronbach’s α = 0.66 in the current study), and the “*orientation toward the host culture*/HC,” e.g., “German traditions now also belong to my life,” with a Cronbach’s α = 0.71 in a previous study (Morawa and Erim, 2014; Cronbach’s α = 0.74 in the current study). Each factor is assessed by 10 items rated on a 7-point scale with the following possible answers: 0 = *does not apply at all*; 1 = *applies seldom*; 2 = *applies less than half the time*; 3 = *applies about half the time*; 4 = *applies more than half the time*; 5 = *applies most of the time*; and 6 = *applies all of the time*.

#### Hopkins Symptom Checklist-25 (HSCL-25)

The Hopkins Symptom Checklist-25 (HSCL-25) is a screening tool to detect anxiety and depression derived from the Hopkins Symptom Checklist (Petermann and Brähler, 2013). It has shown a high Cronbach’s α of 0.92 in previous research (Glaesmera et al., 2014) and has been frequently used to examine samples of refugees (e.g., Schweitzer et al., 2006; Morina et al., 2018). It has shown excellent psychometric properties in research with refugees from many cultures (Mollica et al., 1987; Lavik et al., 2002). The HSCL-25 includes a 15-item subscale for depression (e.g., “crying easily” and “feeling blue”; a Cronbach’s α of 0.91 in the current study). Each item is rated on a Likert scale from 1 to 4 with the following response options: 1 = *not at all*; 2 = *a little*; 3 = *quite a bit*; and 4 = *extremely*. The reference period is the past month.

#### Post-traumatic Diagnostic Scale/Harvard Trauma Questionnaire (PDS/HTQ)

Similar to previous studies assessing refugees (e.g., Morina et al., 2016, 2018), trauma exposure was measured by combining the trauma event lists of the Harvard Trauma Questionnaire with the Post-traumatic Diagnostic Scale to have a scale indexing exposure to traumatic events associated with refugee experiences. The Post-traumatic Diagnostic Scale (PDS) has shown a Cronbach’s α of 0.92 (Foa et al., 1997) and has already shown validity in research with refugees (Norris and Aroian, 2008) and the Harvard Trauma Questionnaire (HTQ; Maercker and Bromberger, 2005, original version with a Cronbach’s α of 0.90; Mollica et al., 1992), has also been shown to be useful in refugee research (Shoeb et al., 2007). This checklist concerning the assessment of traumatic events consists of 25 items (e.g., “being close to death” and “lack of food or water”; a Cronbach’s α of 0.91 in the current study). The items are rated on a 5-point scale with the following options: *happened to me*; *witnessed it*; *have heard of it*; *part of my job*; or *neither nor*. The number of traumatic events was derived from the sum score of the items that were rated with “*happened to me*” and “*witnessed it*,” since witnessing traumatic events is known to exert similar effects on psychological symptoms as experiencing them (Robinson and Larson, 2010; Tierens et al., 2012; Pitts et al., 2014) according to the criteria for a PTSD diagnosis according to the DSM-IV (American Psychiatric Association, 1994). According to these criteria, an event for which a person was an eyewitness and an event that “happened to the person” are both classified as crucial incidents.

### Data Analysis

Missing data were checked using Little’s MCAR test, indicating that data were missing completely at random. Missing values were replaced via multiple imputation using a fully conditional specification (Liu and De, 2015). Studentized residuals were calculated and tested for significance to detect potential outliers as suggested by Zakaria et al. (2014). No outliers were detected. A convergence to normality can be assumed since the sample size is *n* > 30 (Kwak and Kim, 2017). Partial plots were examined for every predictor variable in the regression model to assess linearity. None of the partial plots showed a non-linear relationship between the predictor and the outcome variable. Residual plots showed a normal distribution and homoscedasticity of the residuals. All predictor variables were *z*-standardized to allow an interpretation of the main effects and the interaction term as well as the generation of standardized betas. The proposed models were analyzed using the psych-Package (Revelle, 2018) in R (R Core Team, 2017).

## Results

Table 1, summarizes the descriptive statistics for all measures. *Orientation toward the host culture* was negatively correlated with depressive symptoms (*r* = −0.23, *p* = 0.024). A significant association was not observed between *orientation toward the culture of origin* and depressive symptoms (*r* = −0.08, *p* = 0.461). Furthermore, the number of traumatic events experienced significantly correlated with depression, *r* = 0.31, *p* = 0.002.

**TABLE 1:** Summary of the descriptive statistics for the overall sample of women.

	*M*	*SD*
Traumatic events (PDS/HTQ)	7.81	5.52
Orientation toward the culture of origin (FRACC)	35.43	10.27
Orientation toward the host culture (FRACC)	39.40	10.09
Depression (HSCL-25)	2.37	0.77
Age	29.28	8.71
Time	11.93	6.78

**N* = *98.**

The correlations between traumatic experiences and *orientation toward the culture of origin* and *orientation toward the host culture* were non-significant (OC: *r* = 0.02, *p* = 0.844; HC: *r* = −0.07, *p* = 0.525) (Table 2).

**TABLE 2:** Correlations of variables used in the analyses.

	1	2	3	4	5
(1) Depressive symptoms					
(2) Traumatic events	0.31^∗∗^				
(3) Orientation toward the host culture	−0.23^∗^	–0.07			
(4) Orientation toward the culture of origin	–0.08	0.02	−0.43^∗∗^		
(5) Age	0.13	0.18	–0.09	–0.09	
(6) Time in country	0.15	0.28^∗^	–0.06	0.10	–0.02

Values are Pearson’s correlation coefficients. **p < 0.01, *p < 0.05.

To control for possible confounding of religion and ethnicity with cultural orientation, we compared differences in depression between Christian (*n* = 14) and Muslim (*n* = 73) participants. The other religious groups were too small to draw statistically meaningful conclusions (*n* ≤ 5). A *t*-test indicated no significant difference in depression between both groups (*t* = −1.02, *p* = 0.323).

Differences between ethnic subgroups were determined by comparing the means in depression scores between women from Afghanistan, Syria and Eritrea. The other ethnic groups were too small to draw statistically meaningful conclusions (*n* ≤ 9). An ANOVA, however, revealed no significant differences in depression scores between ethnic groups [*F*(2,68) = 0.58, *p* = 0.561].

We used a model with the FRACC subscales *orientation toward the host culture* and *orientation toward the culture of origin*, as well as their interaction, as moderators on the effect of number of traumatic events on depression to investigate our primary hypothesis. The interaction of both dimensions was included in our model to account for the association of the *orientation toward the host culture* and the *culture of origin*, thereby investigating the possible effect of acculturation strategies. We also used *age* and *time since arrival in the host country* as moderating variables to control for the effect of covariates.

A regression analysis resulted in a significant model [*F*(11,86) = 2.87, *p* = 0.003] that explained 26.85% of the variance in depression, representing a large effect size (*Cohen’s f^2^* = 0.37). The main effects of number of traumatic events (β = 0.20, *p* = 0.035) and *orientation toward the host culture* were also significant (β = −0.23, *p* = 0.006). The main effect of the *orientation toward the culture of origin* (β = −0.16, *p* = 0.066) was not significant.

Furthermore, contrary to our expectations, a significant interaction between the number of traumatic events and *orientation toward the host culture* (β = −0.04, *p* = 0.603), or between the number of traumatic events and *orientation toward the culture of origin* (β = −0.08, *p* = 0.309) was not observed, indicating no moderating effect of both cultural orientations on the relationship between the number of traumatic events and depression. The interaction between *orientation toward the culture of origin* and *orientation toward the host culture* (β = 0.14, *p* = 0.083) was not significant. Additionally, the three-way interaction between the number of traumatic events, *orientation toward the host culture* and *orientation toward the culture of origin* (β = −0.04, *p* = 0.612) was not statistically significant, indicating no moderating effect of acculturation strategies on the relationship of the number of traumatic events on depression. There were no significant effects of age or time on depression, and moderating effects of these covariates on the effect of traumatic events on depressive symptoms were not detected.

## Discussion

Consistent with previous studies (Dulin and Passmore, 2010; Price and van Stolk-Cooke, 2015), we found that traumatic events are positively related to depressive symptoms. The more traumatic events the women experienced, the more depressive symptoms they reported. This effect was significant even after controlling for the time spent in host country and participants age.

However, the results of our study also indicate that neither *orientation toward the culture of origin* nor *orientation toward the host culture* significantly moderate the relationship between the number of traumatic events and depression, indicated by non-significant interactions. Furthermore, the relationship between the number of traumatic events and depression was also not shown to be moderated by the interaction of *orientations toward host culture* and *culture of origin*, reflecting acculturation strategies.

Consistent with previous findings (Ji and Duan, 2006; Meghani and Harvey, 2016), *orientation toward the host culture* was negatively related to depression scores indicating higher *orientation toward host culture* goes along with lower depression scores. Since there was no significant interaction with the number of traumatic events, the effect of the cultural orientation appears to be independent of the number of traumatic events.

The *orientation toward the culture of origin* did not correlate significantly with depression, and the interaction of the *orientation toward the culture of origin* and the number of traumatic events was not significant in our model. This finding contradicts our hypothesis and previously reported research showing an association between a cultural attachment to culture of origin and resilience (Hong et al., 2013).

However, since both, the main effect of the *orientation toward the culture of origin* (β = −0.16, *p* = 0.066) and the effect of the interaction between the *orientation toward the host culture* and the *orientation toward the culture of origin* (β = 0.14, *p* = 0.083) showed considerable trends toward statistical significance, the role of *orientation toward the culture of origin* warrants further investigation. The trend of the main effect points into the direction that higher *orientation toward the culture of origin* might go along with lower depressive symptoms. Thereby, the statistical power in our study may have concealed the protective effect of *orientation toward the culture of origin* against depression. Furthermore, our refugee sample was heterogeneous with regard to countries of origin, which might also blur the relationship between cultural *orientation toward the culture of origin* and depression.

Taken together, the results do not confirm our hypothesis that the relationship between the number of traumatic events and depression varies in female refugees depending on acculturation strategies. Still, we found the number of traumatic events and depression to be related, as well as the *orientation toward the host culture* and depression are.

Furthermore, a number of limitations of this study deserve to be addressed. First, it should be noted that our sample of women was quite heterogeneous with respect to nationality, culture and religion. This heterogeneity might have concealed some culture-specific aspects (Hwang et al., 2008). Secondly, acculturation is a complex process (e.g., Ward and Kus, 2012; Consedine et al., 2014), influenced by numerous geopolitical, social and psychological factors. Therefore, our model is not capable of representing all facets of acculturation like for example emotions or cognitions in detail. Third, a limitation of this study concerns the use of a sum score for traumatic events, thereby merging different types of trauma. Although several studies have shown that the number of traumatic events is a significant predictor of subsequent development of psychopathology (Tanskanen et al., 2004; Heeren et al., 2012), other studies have shown that exposure to interpersonal trauma results in higher level of psychiatric symptoms than non-interpersonal trauma (Hexel and Sonneck, 2002; Lilly et al., 2011). A discrimination of interpersonal vs. non-interpersonal trauma event, may facilitate the detection of associations, in particular with regard to potential moderation effects of acculturation on depression. We were not able to separate different types of trauma because of our small sample size, but this should be addressed in further studies since for example concepts like diminished world assumptions were shown to mediate the relationship of trauma exposure and depression only concerning interpersonal forms of trauma (Lilly et al., 2011). Besides differences in pre-migration trauma, post-migration stress is another facet. For many refugees in Germany, the long-term perspective of permanent residence is insecure. In addition, public discussion about asylum-seekers is polarized, resulting in recurrent discrimination in daily life. These post-migration difficulties are associated with considerable distress and contribute to the maintenance of psychological symptoms such as depression and PTSD (Li et al., 2016; Schock et al., 2016; Miller and Rasmussen, 2017). Fourth, we used instruments that reflect Western concepts of psychopathology. However, the well-known cultural influence on the conceptualization of depression, trauma and acculturation may have impaired the validity of self-report in our sample since it consists of women of different nationalities. We can also not eliminate the problem that there might be a negative evaluation bias influencing the reports. Finally, the cross-sectional design of our study weakens the validity of conclusions regarding causal relationships. The direction of the effect remains unclear. For instance, besides the possible protective effect of *orientation toward the host culture* on depression, depression may also impede the adaptation toward the host culture (Wickrama et al., 2002; Schick et al., 2016).

Further research should address female refugees who have resided in their respective host country for a longer time and have lived under circumstances that have enabled them to be in contact with the host culture in the ways they choose. Living in shared accommodations might conceal the possible effect of the host culture. A clear differentiation between traumatic events associated with the culture of origin and events associated with the host culture would potentially clarify the effect of the cultural association of the trauma. The effect of the severity of traumatic events should also be observed. Additionally, a longitudinal investigation would provide insights into the causal relationships between trauma, acculturation and depression.

Still, there are important implications for receiving countries. Since social integration and mental health seem to be interrelated, enabling female refugees’ to participate in society, economy and culture of the host country may contribute significantly to the prevention of mental disorders in this vulnerable subpopulation. Their participation might either buffer or ease depressive symptoms or participation has to be facilitated since it is particularly difficult for women with depressive symptoms, a condition experienced by a high number of female refugees (Kröger et al., 2016).

Some important clinical implications can be drawn from our work as well. Psychological treatments for refugees with depression should address the possible effect of the acculturation style. Additional knowledge of these effects might help clinicians work with refugees to gain a deeper understanding of their symptoms and thereby improve their psychotherapeutic work. Furthermore, to classify the effect of traumatic experiences, the effect of acculturation should receive consideration.

Overall, our data support the view that independently from the amount of traumatization, *orientation toward the host culture* is associated with lower levels of depression. The results of our study also indicate, that the role of *orientation toward the culture of origin* as a possible protective factor against depression requires further investigation.

## Data Availability Statement

The datasets generated for this study are available on request to the corresponding author.

## Ethics Statement

The studies involving human participants were reviewed and approved by Frankfurt University Hospital, as well as Ethics Committee 1 of Charité Berlin. The patients/participants provided their written informed consent to participate in this study.

## Author Contributions

AS wrote the manuscript with support from US, JG, MS-O, SB, and JJ. US directed the project in Frankfurt. US, JG, and AS carried out the project in Frankfurt. MS-O designed and directed the project in Berlin. JJ coordinated the project in Berlin. All authors discussed the results and contributed to the final manuscript.

## Conflict of Interest

The authors declare that the research was conducted in the absence of any commercial or financial relationships that could be construed as a potential conflict of interest.
